# Don’t judge the book by its cover….

**DOI:** 10.1007/s40620-021-00980-9

**Published:** 2021-03-08

**Authors:** Ettore Pasquinucci, Vittoria Esposito, Giuseppe Sileno, Marco Colucci, Marta Arazzi, Ciro Esposito

**Affiliations:** 1 Nephrology and Dialysis Unit, ICS Maugeri SpA SB, Pavia, Italy; 2 Dept of Internal Medicine and Medical Therapy, University of Pavia, Via Maugeri 10, 27100 Pavia, Italy

**Keywords:** Calcium channel blockers, Peritoneal dialysis, Cloudy effluent, Peritonitis, CKD

**Fig. 1:**
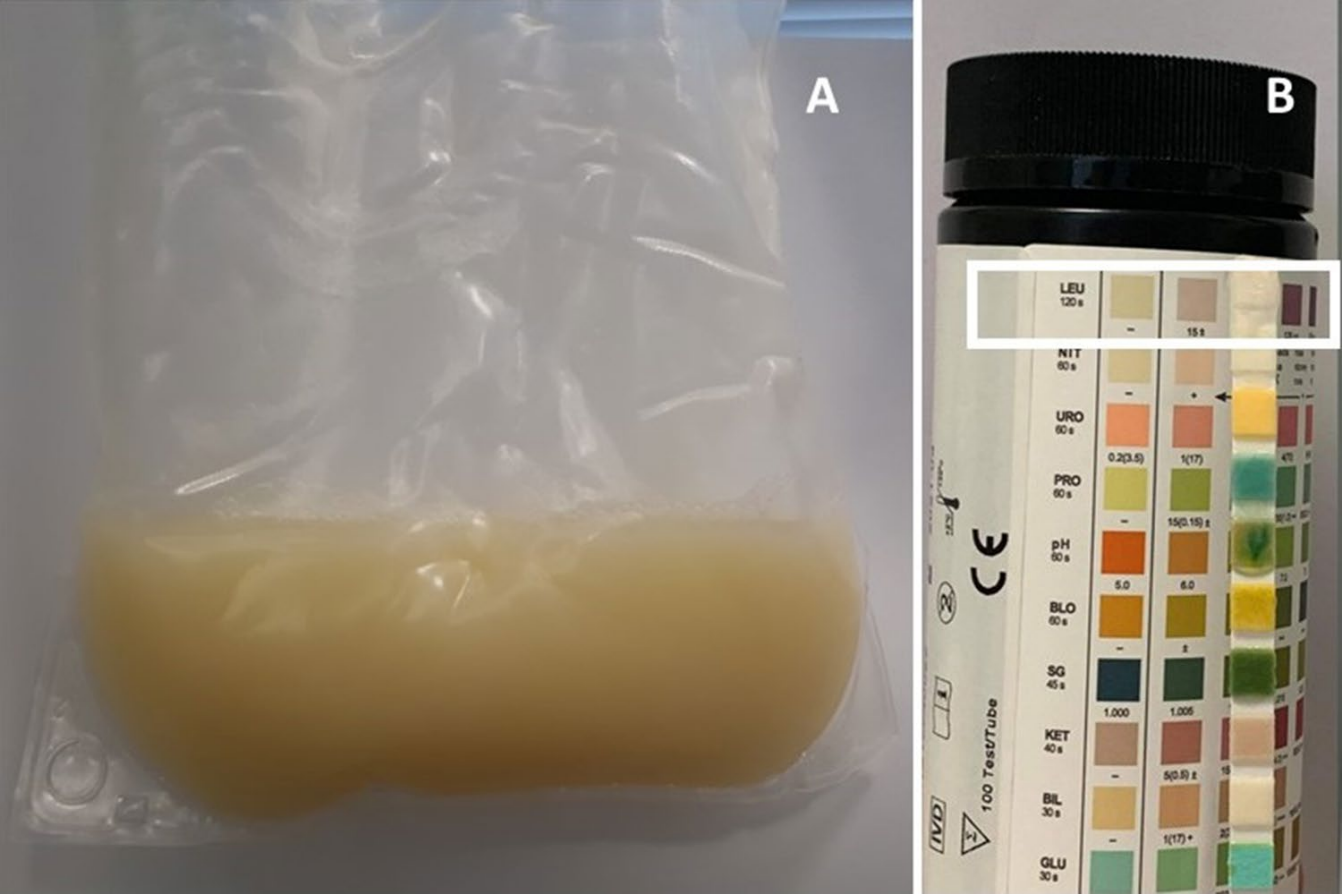
(**A**) Appearance of the peritoneal effluent. (**B**) Peritoneal effluent stick indicating absence of leukocytes (inset)

A 61-year-old caucasian male patient undergoing peritoneal dialysis, contacted our Centre, frightened about the appearance of cloudy peritoneal effluent. His medical history was significant for chronic kidney disease secondary to AL systemic amyloidosis, ischemic heart disease, hypertension, dyslipidaemia. On admission the peritoneal fluid was markedly cloudy and yellowish (Fig. 1a). The patient reported no change in the peritoneal ultrafiltration. A stick of the effluent fluid was negative for leukocytes (Fig. 1b) and white cell count on peritoneal effluent showed 30 cells/mmc. Peritoneal effluent and catheter exite site cultures and lab tests were performed. Even though the clinical suspicion of peritonitis was low, we eventually loaded the patient with a 2000 ml dwell cointaining glucose 1.36% heparin 2000 IU, gentamicin 16 mg and cefazolin 1000 mg. The results of the exams (Table 1) were all negative. During the medical examination the patient revealed that due to poor blood pressure control he had started taking lercanidipine (20 mg/day) as suggested by his family doctor two days before the present admission. Peritoneal effluent lipids profile showed triglycerides 513 mg/dl, cholesterol 20 mg/dl, LDL 4 mg/dl. A diagnosis of lercanidipine-induced chyloperitoneum was made. Lercanidipine treatment was stopped and the peritoneal effluent fluid cleared in 24 h.

**Table 1:** Results of blood and peritoneal effluent lab tests

Parameter	Result	Reference range
Creatinine	8.99 mg/dl	0.7–1.2
Urea	124 mg/dl	18–55
WBC	7.55 10^9^/l	4–10
Hb	12.5 g/dl	14–18
PLT	251 10^9^/l	150–450
C-reactive protein	< 0.1 mg/dl	0–0.5
Total plasma cholesterol	127 mg/dl	0–199
Plasma LDL	64 mg/dl	0–99
Plasma triglycerides	97 mg/dl	0–149
Peritoneal effluent WBC	0.03 10^9^/l	
Peritoneal total cholesterol	20 mg/dl	
Peritoneal LDL	4 mg/dl	
Peritoneal triglycerides	513 mg/dl	

In patients undergoing peritoneal dialysis the appearance of cloudy peritoneal effluent warrants the exclusion of infectious peritonitis. However, there are other rarer causes of opalescent peritoneal effluent [1]. Chyloperitoneum is defined by milky appearance of peritoneal fluid with elevated triglycerides content (usually > 200 mg/dl). This condition is generally caused by lymphatics obstruction secondary to trauma, cancer, cirrhosis, infections. Among iatrogenic causes calcium channel blockers and aliskiren have been implicated. Chyloperitoneum secondary to calcium channel blockers (CCB) has been described both in patients undergoing peritoneal dialysis and in general population [2]. Although our patient is Caucasian, the majority of cases of chyloperitoneum have been described in Asian patients and this may indicate an ethnic predisposition. No gender susceptibility has been found. The underlying mechanism is unclear but it is likely related to lymphatics dysfunction in triglycerides disposal through inhibition of smooth muscle activity of lymphatic vessels by CCB. Two recent systematic reviews on CCB-associated chyloperitoneum found that lercanidipine is the most common causative agent, with appearance of cloudy peritoneal dialysate within four days from drug initiation [3, 4]. Usually peritoneal fluid clears within 24–48 h after drug discontinuation. Although some reports have already been published and the reported incidence ranges from 13 to 57% and it is 12.5% in our center, CCB induced cloudy sterile effluent peritoneal fluid remains an unknown entity to many nephrologists. Furthermore patients on peritoneal dialysis are not always made aware of CCB as a cause of cloudy sterile peritoneal fluid and therefore the sight of the cloudy effluent generates a lot of distress [5]. Nephrologists awareness of CCB induced Chyloperitoneum may help rule out a diagnosis of peritonitis in asymptomatic patients presenting with sterile cloudy fluid having recently started CCB treatment, avoiding stressful situations to the patient and limiting invasive procedures and treatments.
